# Emergency primary anastomosis with or without intraoperative colonic lavage following sigmoidectomy in sigmoid volvulus: 175-patient experience in a tertiary referral hospital

**DOI:** 10.12669/pjms.41.2.11399

**Published:** 2025-02

**Authors:** Necip Altundas, Rifat Peksoz, Esra Disci, Sabri Selcuk Atamanalp

**Affiliations:** 1 Necip Altundas, MD Assistant Professor, Department of General Surgery, Faculty of Medicine, Ataturk University, Erzurum, Turkiye; 2 Esra Disci, MD Associate Professor, Department of General Surgery, Faculty of Medicine, Ataturk University, Erzurum, Turkiye; 3 Rifat Peksoz, MD Associate Professor, Department of General Surgery, Faculty of Medicine, Ataturk University, Erzurum, Turkiye; 4 Sabri Selcuk Atamanalp, MD Professor, Department of General Surgery, Faculty of Medicine, Ataturk University, Erzurum, Turkiye

**Keywords:** Intestinal continuity, Intraoperative colonic lavage, Primary anastomosis, Sigmoid volvulus, Sigmoidectomy

## Abstract

**Objectives:**

Emergency primary anastomosis following sigmoidectomy is one of the main treatment options in sigmoid volvulus (SV). However, during this procedure, the role of intraoperative colonic lavage (ICL) is controversial. Our aim was to evaluate the role of ICL in 175-patient SV series.

**Methods:**

In Ataturk University Faculty of Medicine Department of General Surgery, ICL was applied in 76 cases (43.4%), while it was not used in the remained 99 patients (56.6%) in 58.5-year period. In a partial retrospective (first 20 years, from June 1966 to June 1986) and prospective (later 38.5 years, from June 1986 to December 2024) evaluation, some preoperative, operative, and postoperative findings were utilized.

**Results:**

As preoperative data, mean age (56.4 years vs. 57.1 years, P>0.05), male/female ratio (4.8 vs. 4.2, P>0.05), and rate of shock (5.3% vs. 6.1%, P>0.05) were statistically similar in both groups, while mean ASA score (3.1 vs. 2.9, P<0.05) was significantly lower in ICL group. Among operative findings, rates of bowel gangrene (67.1% vs. 63.6%, P>0.05) and perforation (1.3% vs. 1.0%, P>0.05) were statistically similar in both groups, while mean operation time (205.4 minutes vs. 176.8 minutes, P<0.005) was significantly longer in ICL group. As surgical outcomes, rates of mortality (13.2% vs 12.1%, P>0.05) and morbidity (39.5% vs. 28.3%, P>0.05) were statistically similar in both groups, while mean hospitalization time (14.7 days vs. 9.4 days, P<0.001) was significantly longer and mean cost (3,455.4 USD vs. 2,752.1 USD, P<0.001) was significantly higher in ICL group.

**Conclusion:**

When compared with that of primary anastomosis with ICL, primary anastomosis without ICL provided shorter operation and hospitalization times, and less cost in addition to similar mortality and morbidity rates in the emergency treatment of SV.

## INTRODUCTION

In sigmoid volvulus (SV), emergency sigmoidectomy is the unique treatment choice in the presence of bowel gangrene.^1^ Similarly, it is a good solution in some selected SV cases with viable bowel.^2^ Following sigmoidectomy, primary anastomosis is the most attractive option in the restoration of bowel continuity.^3^-^5^ However, the role of intraoperative colonic lavage (ICL) before emergency primary anastomosis is controversial.^6^,^7^ In this study, to evaluate the role of ICL in the emergency sigmoidectomy and primary anastomosis in SV, we investigated the surgical outcomes of 175 patients (16.1%) among total 1,088 SV cases treated over 58.5 years (from June 1966 to December 2024). This series involves the largest single-center SV data over the world.^8^,^9^

## METHODS

Among total 1,088 patients treated in Ataturk University, Faculty of Medicine, Department of General Surgery, a retrospective evaluation was made in the clinical records of 612 cases (56.3%), who were treated in the first 20 years (from June 1966 to June 1986). The remaining 476 patients (43.8%), who were treated in the later 38.5 years (from June 1986 to December 2024), were evaluated prospectively. In patients treated with emergency sigmoidectomy and primary anastomosis, we recorded preoperative data including age, gender, shock, and American Society of Anesthesiologists (ASA) score. We also noted operative findings such as bowel gangrene, perforation, risky bowel (borderline ischemia, edema, or different diameter in bowel ends), and operation time. Finally, we inscrolled early outcomes including mortality, morbidity, hospitalization time and cost.

Inclusion criterion of this study was the treatment of SV with emergency primary anastomosis with or without intraoperative colonic lavage following sigmoidectomy. There was no exclusion criterion for above-mentioned surgery group except for other treatment options of SV including nonsurgical or non-resectional methods. In emergency surgery of SV, following sigmoidectomy, we used primary anastomosis with or without ICL. In ICL, we used 1,000-3,000 cc. isotonic sodium chloride solution via tube cecostomy from the appendiceal base following the resection of the distal part of the sigmoid colon. Cecostomy tube placed the appendiceal base was removed 10-14 days later.

### Statistical Analysis:

In the statistical comparison of the outcomes of primary anastomosis with or without ICL, analyses were performed with IBM SPSS 20 statistical analysis programme. We used mean, standard deviation, percentage, and number in the presentation of the data. Shapiro Wilk test, Kolmogorov Smirnov test, Q-Q plot, skewness and kurtosis were used in the analysis of normal distribution of continuous variables. The comparison of independent groups was made by using Independent Samples t test in the presence of normal distribution, while Mann Whitney u test was preferred in the absence of it. Pearson Chi-square test was used for 2x2 comparisons between categorical variables. Statistical significance level was set as p<0.05.

### Ethical Approval:

For this study, we procured informed consents from the patients and we obtained ethical approval from the Ethical Committee of Ataturk University Faculty of Medicine (Number: 68, Date: February 21, 2024).

## RESULTS

Among total 175 SV patients treated with emergency sigmoidectomy and primary anastomosis, we used ICL in 76 cases (ICL group, 43.4%), while we did not apply ICL in the remained 99 patients (non-ICL group, 56.6%). Data on our series and related statistical analyses are given in Table-I. In the consideration of the preoperative findings, mean age (56.4 years vs. 57.1 years, P>0.05), male/female ratio (4.8 vs. 4.2, P>0.05), and rate of shock (5.3% vs. 6.1%, P>0.05) were statistically similar in ICL and non-ICL groups, while mean ASA score (3.1 vs. 2.9, P<0.05) was significantly lower in ICL group. As operative findings, rates of bowel gangrene (67.1% vs. 63.6%, P>0.05), perforation (1.3% vs. 1.0%, P>0.05), and risky bowel (5.3% vs. 4.0%, P>0.05) were statistically similar in ICL and non-ICL groups, while mean operation time (205.4 minutes vs. 176.8 minutes, P<0.005) was significantly longer in ICL group.

**Table-I:** Preoperative, operative, and postoperative characteristics of the patients with sigmoid volvulus treated with emergency sigmoidectomy and primary anastomosis with or without intraoperative colonic lavage.

Parameter	With ICL	Without ICL	Statistical analysis, P
Total	76	99	-
Age	56.4±13.9	57.1±14.6	Independent Samples t test 0.748
Gender (male/female) %	63/13	80/19	Chi-Square test
	4.8	4.2	0.723
Shock %	4	6	Chi-Square test
	5.3	6.1	0.822
ASA score	3.1±0.7	2.9±0.5	Independent Samples t test 0.028
Bowel gangrene %	51	63	Chi-Square test
	67.1	63.6	0.633
Bowel perforation %	1	1	Chi-Square test
	1.3	1.0	0.850
Risky bowel^a^%	4	4	Chi-Square test
	5.3	4.0	0.701
Operation time (minutes)	205.4±67.1	176.8±58.3	Independent Samples t test 0.003
Mortality %	10	12	Chi-Square test
	13.2	12.1	0.837
Morbidity %	30	28	Chi-Square test
	39.5	28.3	0.119
Hospitalization time (day)	14.7±7.9	9.4±5.7	Independent Samples t test <0.001
Cost (USD)	3.455.4±1.016.7	2.752.1±822.0	Independent Samples t test <0.001

ICL: Intraoperative colonic lavage, ASA: American Society of Anesthesiologists, Risky bowel: borderline ischemia, edema, or different diameter in bowel ends.

Among postoperative findings, rates of mortality (13.2% vs 12.1%, P>0.05) and morbidity (39.5% vs. 28.3%, P>0.05) were statistically similar in ICL and non-ICL groups. Wound infection was the most common complication in both groups, while leakage from irrigation site as well as anastomotic leakage were other rare surgery-specific complications in ICL and non-ICL groups, respectively. However, mean hospitalization time (14.7 days vs. 9.4 days, P<0.001) was significantly longer and mean cost (3,455.4 USD vs. 2,752.1 USD, P<0.001) was significantly higher in ICL group.

## DISCUSSION

In sigmoid volvulus (SV), sigmoidectomy with primary anastomosis is the most desirable surgical option, which provides quite good surgical outcomes and prevents SV recurrence.^10^,^11^ In emergency colorectal surgery including SV, preoperative bowel preparation has protective effects against mortality, morbidity including anastomotic leakage, and prolonged hospitalization.^12^ Although ICL with primary anastomosis provides similar advantages when compared with that of stoma and stoma closure operations, its role is controversial when compared with that of primary anastomosis alone.^6^ Prolonged operation time, risk of contamination, and electrolyte imbalance appear as the major disadvantages of ICL.^13^

Most commonly used form of ICL was first introduced by Dudley et al.^14^ in 1980. Although the effects of different forms of ICL on several kinds of colorectal surgery including cancer and diverticular disease are discussed in detail from that day to this, there is no enough data on the role of ICL in the emergency surgical treatment of SV.^15^-^17^ In the present study, we evaluated a relatively large SV series treated with emergency surgery including sigmoidectomy and primary anastomosis with or without ICL. Although the mean ASA scores were statistically higher in ICL group, we demonstrated significantly shorter operation and hospitalization times, and less cost in patients treated without ICL. Additionally, the mortality and morbidity rates were statistically similar in both groups.

As seen in Table-II, among limited data on the role of emergency primary anastomosis with ICL in SV, a relatively large series including 32 patients was reported by Sule and Ajibade.^18^ In this retrospective evaluation, the authors have showed 9.4% of mortality and 31.3% of morbidity rates. In a prospective report by Akdemir et al.^19^ the mortality and morbidity rates were reported as 0.0% and 10.0%, respectively, in a 20-patient population. In this series, the mean operation time was 157.4 minutes and the mean hospitalization time was 14.8 days. On the other hand, total three papers including five cases presented the mortality and morbidity rates as 0.0% and 20.0%, respectively. In above-mentioned reports related to primary anastomosis with ICL in SV, the most common morbidity was wound infection, while leakage from irrigation site was a rare but a surgery-specific problem. On the other hand, prolonged mean operation (mean 35.8-minute of prolongation) and hospitalization (up to mean 31 days) times were other attention-grabbing results.^20^-^22^ In our opinion and experience, colonic irrigation with tube cecostomy is the most effective factor in the prolongation of the operation time in primary anastomosis with ICL. Similarly, waiting for the removal of the cecostomy tube during the hospitalization period is the most important time-consuming factor in the evaluation of the hospitalization time.

**Table-II:** Large-series literature data on primary anastomosis with or without intraoperative colonic lavage following emergency sigmoidectomy in sigmoid volvulus.

Author	Country	Year	Bowel gangrene	ICL	No	Mortality (%)	Morbidity (%)
Akdemir et al.^19^	Turkey	1991	Yes/No	Yes	20	0 (0.0)	2 (10.0)
De and Ghosh.^24^	India	2003	Yes/No	No	197	74 (37.6)	2 (1.0)
Sule and Ajibade.^18^	Nigeria	2011	No	Yes	32	3 (9.4)	10 (31.3)
Traore et al.^25^	Mali	2014	Yes/No	No	149	12 (8.1)	32 (21.5)
Pattanaik et al.^13^	India	2018	Yes/No	No	270	20 (7.4)	44 (16.3)
Dolejs et al.^23^	India	2018	Yes/No	No	440	40 (9.1)	223 (50.7)
Awedew et al.^3^	Ethiopia	2023	Yes	No	301	45 (15.0)	?
Yang et al.^1^	USA	2024	Yes/No	No	895	112 (12.5)	373 (41.7)
Present series	Turkey	2024	Yes/No Yes/No	Yes No	76 99	10 (13.2) 12 (12.1)	30 (39.5) 28 (28.3)

ICL: Intraoperative colonic lavage.

Contrary to primary anastomosis with ICL, a great number of reports are present in the literature on the surgical outcomes of primary anastomosis without ICL in SV, some of which are presented in Table-II. In a multicenter retrospective study, a relatively large series including 895 patients was evaluated by Yang et al.^1^ In this report, the mortality and morbidity rates were reported as 12.5% and 41.7%, respectively, while the mean hospitalization time was reported as 11.3 days. In another 440-case retrospective study, these parameters were reported as 9.1%, 50.7%, and eight days in addition to 93-minute mean operation time by Dolejs et al.^23^ In a meta-analysis by Awedew et al.^3^ the mortality rate was found to be 15.0% in 301-patient series. However, Pattanaik et al.^13^ reported relatively better prognostic data including 7.4% of mortality and 16.3% of morbidity rates in their 270-case retrospective evaluation. Similarly, De and Ghosh^24^ retrospectively demonstrated 1.0% of mortality and 37.6 of morbidity rates with mean 9.8 day-hospitalization time in their 197 patients. Finally, in a 149-case retrospective evaluation reported by Traore et al.^25^ the mortality and morbidity rates were 8.1% and 21.5%, respectively. In patients treated with primary anastomosis without ICL in SV, the wound infection was the most common morbidity, while anastomotic leakage was a rare surgery-specific complication.

Based on our results, in the emergency surgical treatment of SV, primary anastomosis without ICL looks like a relatively better option in comparison to primary anastomosis with ICL due to shorter operation and hospitalization times, and cheaper cost.

### Limitations:

In this study, partial retrospective analysis, nonrandomized sampling, and non-matched statistical evaluation are the main limitations. Although SV is the most common cause of intestinal obstructions in most eastern countries, its incidence is not high all over the world, particularly in western populations. For this reason, prospective randomized considerations necessitate tens of patients and decades. However, matched statistical evaluation of large retrospective series may provide partial advantages.

## CONCLUSIONS

According to our non-matched evaluation including 175 patients with SV, shorter operation and hospitalization times, and less costly appear as the major advantages of primary anastomosis without ICL when compared with that of primary anastomosis with ICL. New prospective randomized clinical studies or matched analyses may give additional information on this subject.

### Authors’ Contribution:

**NA and SSA:** Data collection, literature search, manuscript writing.

**ED and RP:** Data collection, critical analysis and revision of the final draft.

**SSA:** Is responsible and accountable for the accuracy or integrity of the work.
